# Chairside Tomosynthesis for Intraoperative Imaging in Implant Dentistry: Clinical Applications and Workflow Considerations

**DOI:** 10.7759/cureus.115286

**Published:** 2026-08-27

**Authors:** David Hansen, Gregori M Kurtzman

**Affiliations:** 1 Dentistry, Woodland Dental, Salem, USA; 2 General Dentistry and Implantology, Private Practice, Silver Spring, USA

**Keywords:** chairside tomosynthesis, cone beam computed tomography, implant dentistry, intraoperative imaging, three-dimensional imaging

## Abstract

Cone-beam computed tomography (CBCT) has become an important component of implant dentistry by providing three-dimensional visualization for diagnosis and treatment planning. However, once implant surgery begins, obtaining additional three-dimensional imaging may require interruption of the procedure and transportation of the patient to a dedicated imaging system. Chairside tomosynthesis offers an alternative approach by providing focused sectional imaging directly within the operatory.

This article describes the principles, clinical applications, and workflow considerations of chairside tomosynthesis in implant dentistry, with emphasis on its potential role in addressing focused intraoperative imaging questions and supporting real-time clinical decision-making.

Unlike CBCT, which provides comprehensive three-dimensional information for diagnosis and treatment planning, tomosynthesis acquires multiple radiographic projections over a limited arc and reconstructs them into sectional images of a focused region of interest. Potential applications during implant surgery include verification of osteotomy trajectory and implant position, assessment of posterior mandibular implants relative to the inferior alveolar canal, evaluation of anterior maxillary implant positioning and cortical plates, immediate implant placement, sinus augmentation, full-arch rehabilitation, revision procedures, and retrieval of retained or fractured implant components. The mobile Lumos 3DX (Valkyrie Bioscience, Lindon) system permits this focused imaging to be performed chairside, potentially reducing disruption of the surgical workflow and providing additional information when a specific clinical question arises.

Chairside tomosynthesis should be considered complementary to, rather than a replacement for, CBCT. CBCT remains the preferred modality for comprehensive diagnosis and preoperative treatment planning, whereas chairside tomosynthesis may provide focused sectional information when additional visualization is required during implant surgery. By providing three-dimensional imaging directly within the operatory, chairside tomosynthesis may help bridge the intraoperative imaging gap and the need for timely sectional imaging during surgery without interrupting the procedure to transport the patient to a dedicated CBCT system, while preserving procedural continuity. Further clinical investigation is necessary to define appropriate indications and determine its diagnostic accuracy, clinical utility, and effect on treatment outcomes.

## Introduction

Few innovations have influenced implant dentistry as profoundly as cone-beam computed tomography (CBCT). For the first time, clinicians could routinely evaluate bone volume, ridge morphology, vital anatomical structures, restorative space, and implant position in three dimensions before treatment began [1-4]. Modern implant procedures have become more accurate, predictable, and considerably safer because of this technology.

Yet implant surgery itself remains inherently dynamic. Even the most carefully planned procedure may present unexpected findings once treatment is underway. The quality of remaining bone following extraction may differ from what was anticipated. An osteotomy may appear closer to a cortical plate than expected. A sinus membrane may not elevate as planned, or a clinician may simply wish to confirm implant position before proceeding with the next surgical step. These situations do not represent failures of treatment planning; rather, they reflect the reality that surgery is performed in living tissues, where anatomy and clinical conditions cannot always be predicted perfectly.

This creates what may be described as the intraoperative imaging gap. Before surgery, clinicians have access to comprehensive three-dimensional information through CBCT. Once surgery begins, however, obtaining additional three-dimensional imaging often becomes inconvenient. In many practices, acquiring another CBCT scan requires covering the surgical site, transporting the patient to a separate imaging room, obtaining the scan, and then returning to the operatory before treatment can continue. Although this workflow is technically feasible, it interrupts the procedure at precisely the moment when clinical momentum and concentration are most important.

As implant dentistry has evolved, clinicians have increasingly recognized that the imaging requirements during surgery differ from those before surgery. Preoperative planning demands comprehensive anatomical visualization. In contrast, intraoperative imaging often seeks to answer a much narrower question: Is the implant trajectory appropriate? Has adequate clearance from the maxillary sinus or inferior alveolar canal been maintained? Has a fractured prosthetic component been completely retrieved? These focused questions rarely require another comprehensive diagnostic examination. Instead, they require timely access to additional sectional information that can guide the next clinical decision.

Conventional intraoral radiography can provide rapid intraoperative confirmation but remains limited by its two-dimensional representation of three-dimensional anatomy, including superimposition and the inability to directly assess buccolingual relationships. Surgical guides and navigation systems may improve transfer of the preoperative plan to the surgical field, but they do not provide new sectional imaging when unexpected anatomical or procedural questions arise during treatment. These limitations create a potential role for focused three-dimensional imaging that can be obtained directly within the operatory.

Tomosynthesis is a limited-angle imaging technique that acquires multiple radiographic projections and reconstructs them into sectional images of a focused region of interest. Unlike conventional radiography, which compresses three-dimensional anatomy into a two-dimensional image, tomosynthesis reduces anatomical superimposition and provides depth information. Unlike CBCT, which generates a comprehensive volumetric dataset, tomosynthesis is intended to provide focused sectional information from a limited region when a specific clinical question arises.

## Technical report

**Figure 1:**
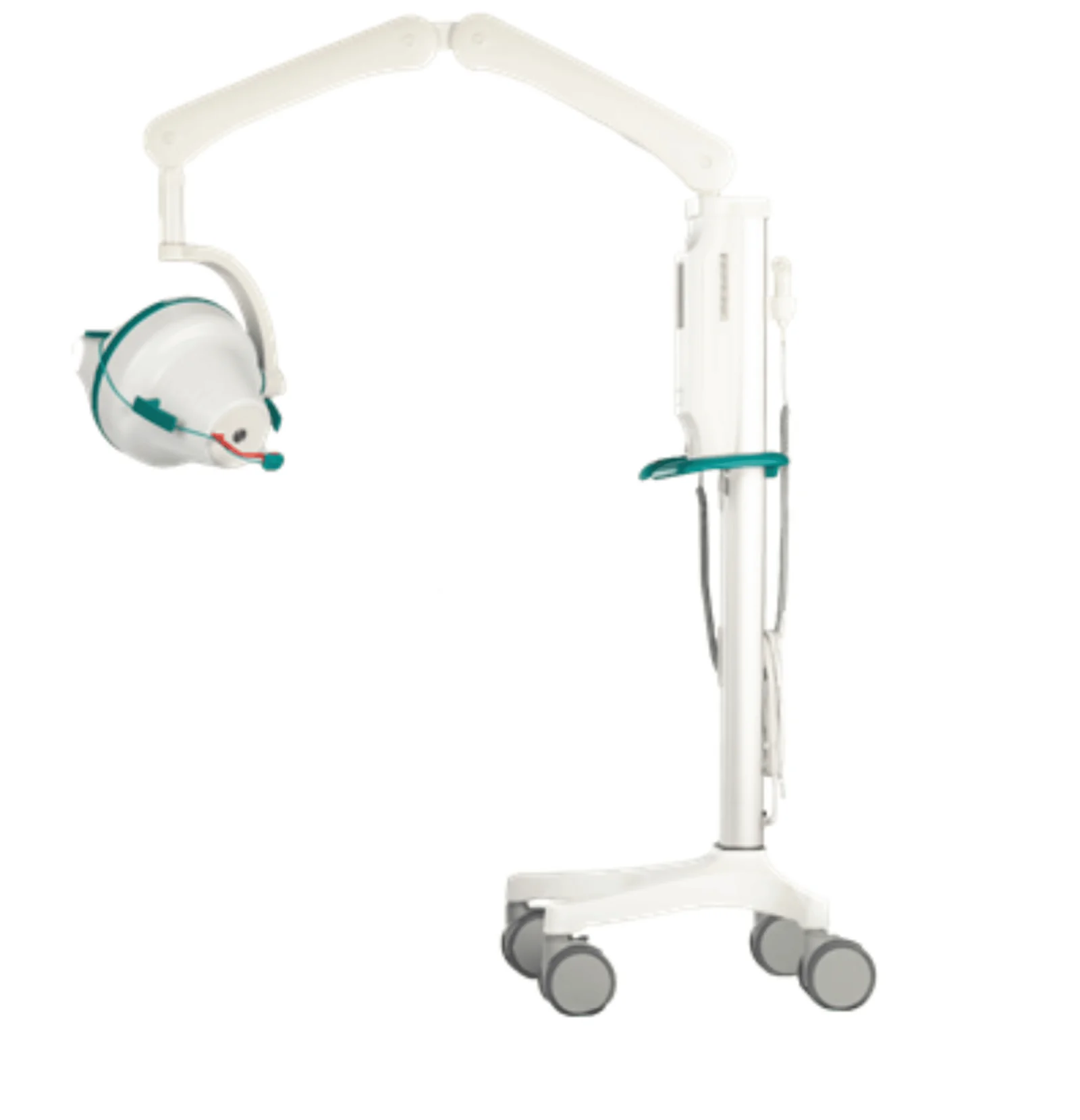
The Lumos 3DX mobile chairside tomosynthesis system. The compact, operatory-based unit acquires focused three-dimensional images at the point of care, allowing supplemental intraoperative imaging without transporting the patient to a dedicated CBCT scanner. Image provided by Valkyrie Bioscience.

**Figure 2:**
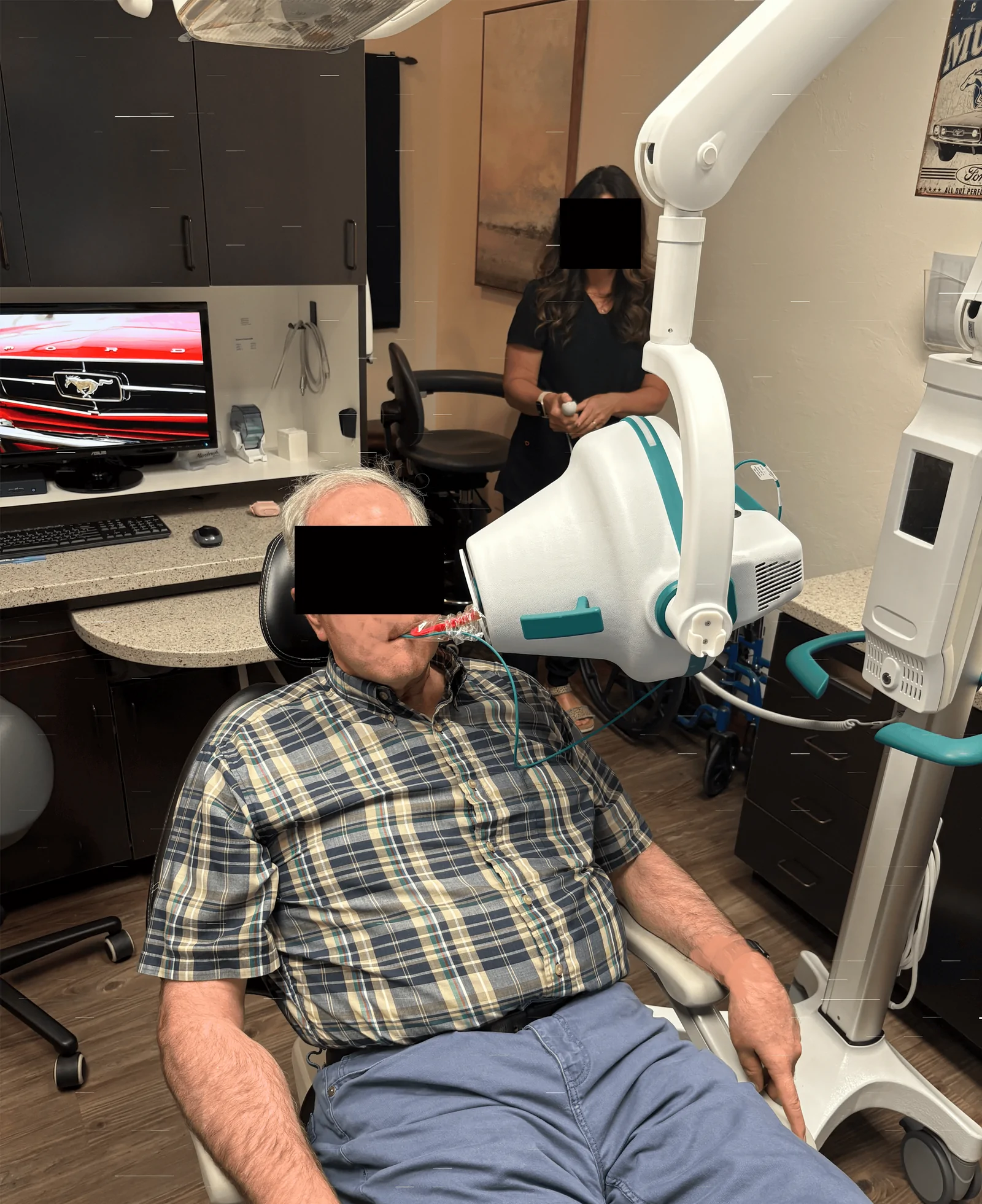
The Lumos 3DX unit being used in the operatory. The picture corresponds to one of the lead author's patients. The face has been obscured to protect the patient's identity, and consent for publication was obtained.

Chairside tomosynthesis addresses the need for focused sectional imaging when a specific clinical question arises during implant surgery. The mobile Lumos 3DX (Valkyrie Bioscience, Lindon) uses limited-angle tomography to generate three-dimensional images from multiple radiographic projections and can be transported between operatories, allowing supplemental imaging to be obtained without moving the patient to a dedicated imaging suite (Figures 1, 2). The FDA 510(k) documentation describes approximately three-second 3D data acquisition [5]. This configuration provides a potential means of obtaining focused sectional information during treatment while minimizing disruption of the surgical workflow. 

CBCT and Lumos 3DX: complementary roles

CBCT remains the foundation of implant imaging. It should continue to be regarded as the primary modality for comprehensive diagnosis, anatomic assessment, prosthetically driven planning, guided surgery design, and evaluation of complex implant sites. The extensive literature supporting CBCT in implant dentistry should not be interpreted as being challenged by chairside tomosynthesis. Instead, Lumos 3DX occupies a different position in the imaging continuum. The complementary roles of both imaging modalities throughout implant treatment are summarized in Table 1.

**Table 1:** Comparison of imaging modalities in implant dentistry. CBCT: cone-beam computed tomography.

Imaging Modality	Primary Purpose	Strengths	Limitations
Conventional radiography	Routine diagnosis and follow-up	Low dose, inexpensive, rapid	Two-dimensional image with anatomical superimposition
Chairside tomosynthesis	Focused intraoperative sectional imaging	Reduces anatomical overlap, obtained chairside, supports real-time decision-making	Limited field of view; not intended for comprehensive diagnosis
CBCT	Comprehensive diagnosis and treatment planning	Complete three-dimensional visualization of anatomical structures	Dedicated scanner, higher cost, may interrupt surgery if repeat imaging is required

Preoperative CBCT answers the question: How should this case be planned? Chairside tomosynthesis helps answer a different question: Am I comfortable proceeding with the next surgical step? That distinction is important. During implant surgery, clinicians frequently require confirmation of one specific relationship rather than a repeat of the entire diagnostic work-up. The goal may be to verify the depth and angulation of an osteotomy, the position of an implant adjacent to the sinus floor, the relationship of a posterior mandibular implant to the nerve canal, or the location of a retained prosthetic component.

The FDA 510(k) summary identifies Lumos 3DX as an extraoral X-ray source with intraoral X-ray detection for producing diagnostic dental radiographs of the teeth, jaw, and other oral structures, with 3D imaging provided via tomosynthesis for adult patients [5]. The same summary describes the system as a 3D dental chairside X-ray system on a mobile stand that uses limited-angle tomography to generate 3D images from multiple projections [5]. These regulatory documents also note approximately three-second 3D data acquisition, a mobile stand configuration, and the ability to use the device for unconscious patients while they remain in the chair rather than requiring transport to another imaging device [5]. Image reconstruction follows acquisition and permits chairside review of the tomosynthetic dataset; however, a specific reconstruction time was not identified in the available regulatory documentation.

What is tomosynthesis?

Most dentists are familiar with the strengths and limitations of both conventional radiography and CBCT. Conventional radiographs remain indispensable because they are inexpensive and rapid and they expose patients to relatively low radiation doses. Their primary limitation, however, is that three-dimensional anatomy is compressed into a single two-dimensional image. As a result, anatomical structures often overlap, obscuring details that may be clinically important.

CBCT addresses this limitation by acquiring hundreds of radiographic projections during a complete rotation around the patient's head [6]. These data are reconstructed into a volumetric dataset that can be viewed in any plane, allowing clinicians to evaluate complex anatomical relationships with exceptional detail. This comprehensive three-dimensional visualization has made CBCT the standard of care for many implant planning procedures.

Tomosynthesis occupies the space between these two imaging modalities [7]. Rather than acquiring a single projection like a conventional radiograph or a complete volumetric dataset like CBCT, the Lumos 3DX acquires 30 radiographic projections spaced every 12 degrees around a circle that forms the base of a cone with the X-ray beam centered on the intra-oral detector for each projection. The X-ray beam traces out a cone with a half-angle of 20 degrees over the approximately 3-second acquisition. These projections are reconstructed into a three-dimensional image dataset containing the focused region of interest that can be viewed in sequential sections similar to CBCT images. By separating anatomy into individual layers, tomosynthesis substantially reduces the superposition that limits conventional radiography while requiring less acquisition time and less complex mobile hardware than a full CBCT system, enabling image acquisition at the chair [8-11]. A familiar medical application of this technology is digital breast tomosynthesis, commonly referred to as 3D mammography. Both use multiple radiographic projections acquired over a limited angular range to reconstruct sectional images, reducing the anatomical superimposition associated with conventional two-dimensional radiography. In dental tomosynthesis, this same general imaging principle is applied to a focused region of the dentoalveolar anatomy. Radiation exposure remains an important consideration when using chairside tomosynthesis, and its use should be clinically justified based on the specific intraoperative question and the principle of limiting radiation exposure to that necessary to obtain the required diagnostic information.

An easy way to visualize the difference is to imagine reading a book. A conventional radiograph resembles looking at every page simultaneously, with all of the text overlapping. CBCT provides the entire book, allowing every page to be examined from any perspective. Tomosynthesis, by comparison, provides access to all the pages in one chapter with some limitations on the perspective. Although it does not reconstruct the complete volume, it allows the clinician to examine the specific section or region most relevant to the clinical question.

This distinction also explains why tomosynthesis should not be viewed as a replacement for CBCT. The two technologies were developed to answer different questions. CBCT is intended for comprehensive diagnosis, treatment planning, and evaluation of complex anatomy. Tomosynthesis provides focused sectional imaging when additional high-resolution information is needed quickly and efficiently. As with other radiographic techniques, tomosynthesis has limitations. Image quality may be affected by patient motion, metallic restorations or implants, and variations in bone density, while the limited acquisition angle may result in residual superimposition or reduced visualization of structures outside the focal region.

Perhaps the greatest contribution of chairside tomosynthesis is not simply the images it produces, but the timing of those images. By providing immediate access to small-volume three-dimensional visualization during surgery, tomosynthesis has the potential to bridge the intraoperative imaging gap between meticulous preoperative planning and confident surgical execution.

Clinical applications of chairside tomosynthesis

The value of any imaging modality depends not only on image quality but also on whether the information obtained changes clinical decision-making. CBCT has become indispensable because it answers comprehensive diagnostic questions before treatment begins. Chairside tomosynthesis addresses a different need by providing focused sectional information during surgery when new questions arise.

Although routine implant procedures often proceed exactly as planned, many cases involve situations in which additional confirmation may improve clinician confidence before moving to the next surgical step. The ability to obtain this information without interrupting treatment represents one of the primary advantages of chairside tomosynthesis.

Implant placement and osteotomy verification

Accurate implant positioning depends on maintaining the planned trajectory while respecting surrounding anatomical structures. During placement, tactile feedback occasionally suggests that the osteotomy may not be following the anticipated path. In other situations, dense cortical bone or limited visibility may create uncertainty regarding angulation or depth. Situations in which supplemental sectional imaging may be particularly useful include dense bone or limited surgical visibility, proximity to the inferior alveolar canal, maxillary sinus, adjacent teeth, or cortical plates, narrow ridges, and sites adjacent to previously placed implants.

Chairside tomosynthesis allows the clinician to obtain focused three-dimensional images that verify implant trajectory before final seating. Rather than replacing preoperative planning, these images provide confirmation that the surgical plan is being executed as intended.

Chairside tomosynthesis may be used to verify osteotomy trajectory and final implant position in situations where tactile feedback or anatomical limitations create uncertainty. Rather than relying solely on postoperative imaging, intraoperative confirmation may allow adjustments before treatment is completed.

Posterior implant placement in the mandible

**Figure 3:**
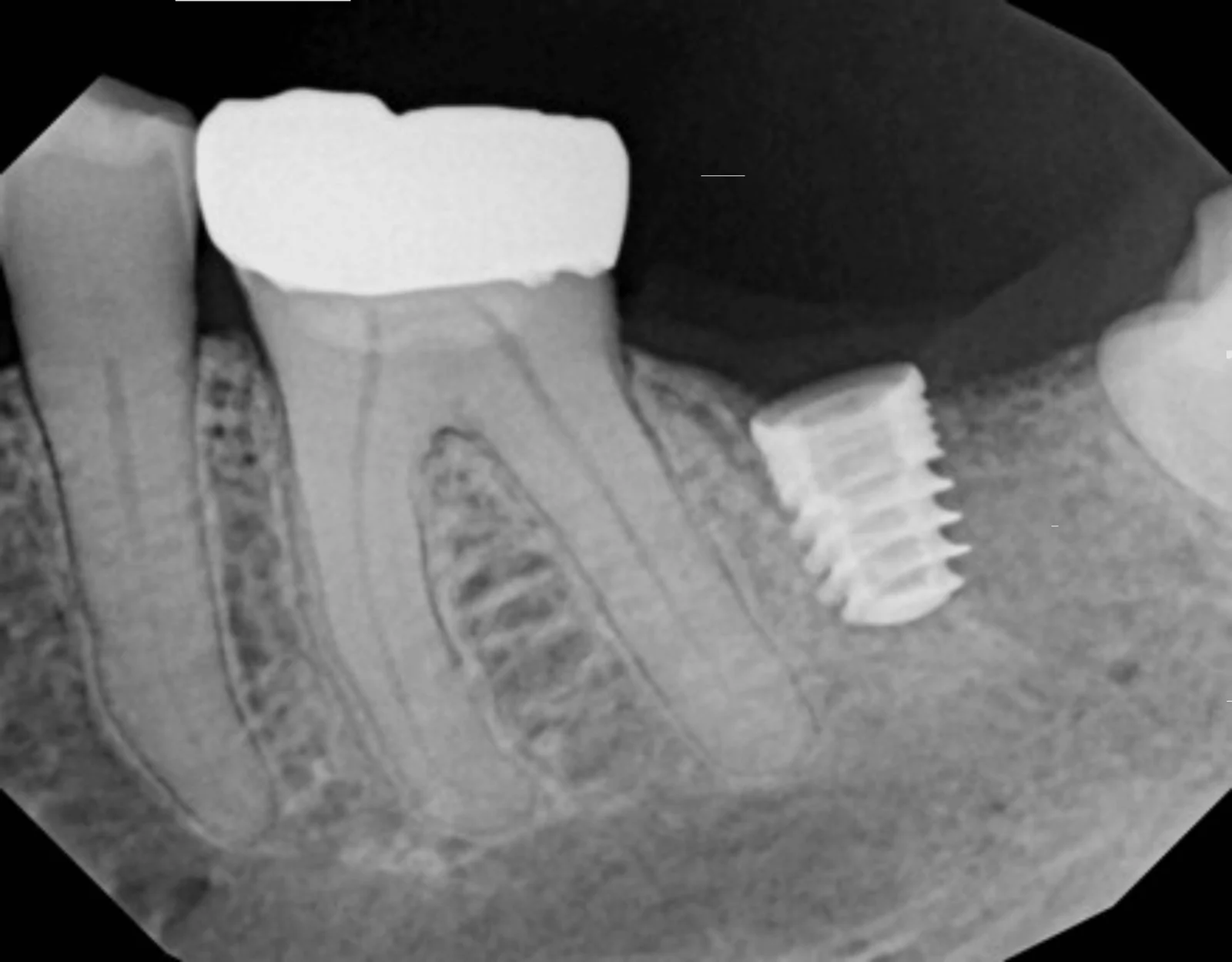
Conventional periapical radiograph following placement of a mandibular implant. While the radiograph demonstrates the implant position relative to the adjacent tooth and crestal bone, it cannot accurately evaluate the buccolingual angulation of the implant or its relationship to the inferior alveolar canal.

Conventional periapical radiographs remain an important component of implant therapy, as they confirm the mesiodistal position of the implant relative to adjacent teeth and crestal bone (Figure 3). However, because they are two-dimensional, they cannot accurately evaluate the buccolingual angulation of the implant or its relationship to the inferior alveolar canal. As a result, the clinician must rely on preoperative CBCT and surgical experience to assess the implant's three-dimensional position.

**Figure 4:**
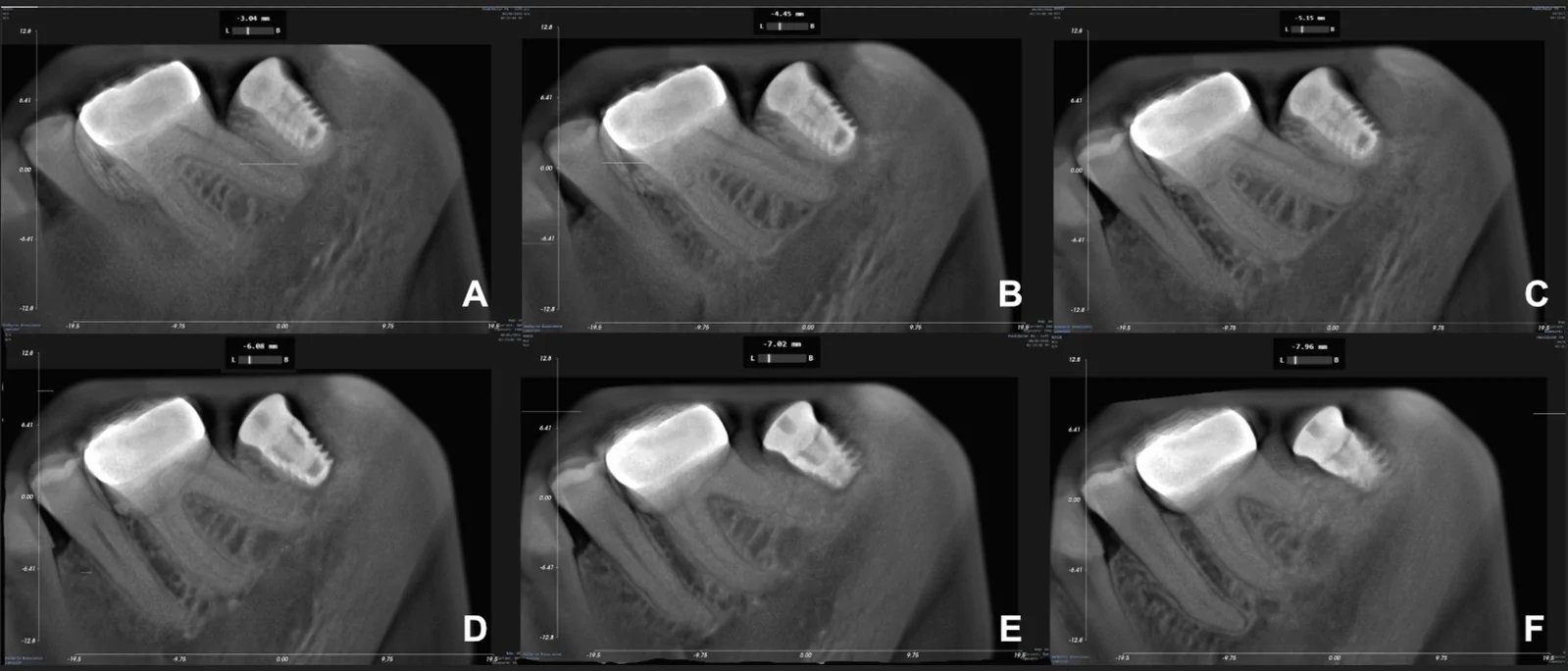
Sequential cross-sectional tomosynthesis images of the posterior mandibular implant site obtained with the Lumos 3DX. The numerical values displayed on the images identify the relative position of the reconstructed sections within the tomosynthetic dataset. Viewing the sequential sections allows assessment of implant position in the buccolingual dimension and demonstrates its relationship to the surrounding cortical bone and inferior alveolar canal, information that cannot be directly determined from the conventional two-dimensional periapical image.

Chairside tomosynthesis with the Lumos 3DX supplements the conventional periapical radiograph by providing sequential cross-sectional views through the implant site (Figure 4). The periapical radiograph demonstrates the mesiodistal position of the implant and its projected relationship to the inferior alveolar canal, but it compresses the buccolingual dimension into a two-dimensional image. In contrast, the tomosynthetic slices allow the implant to be followed through the buccolingual dimension, providing additional information regarding implant angulation, its position within the alveolar housing, and its three-dimensional relationship to the inferior alveolar canal. This distinction is clinically important because an implant that appears appropriately positioned on the periapical radiograph may still be positioned too far buccally or lingually, a relationship that cannot be determined from the two-dimensional image alone. If an unfavorable position is identified intraoperatively, corrective treatment may be considered before completion of the procedure.

Anterior maxillary implant placement

**Figure 5:**
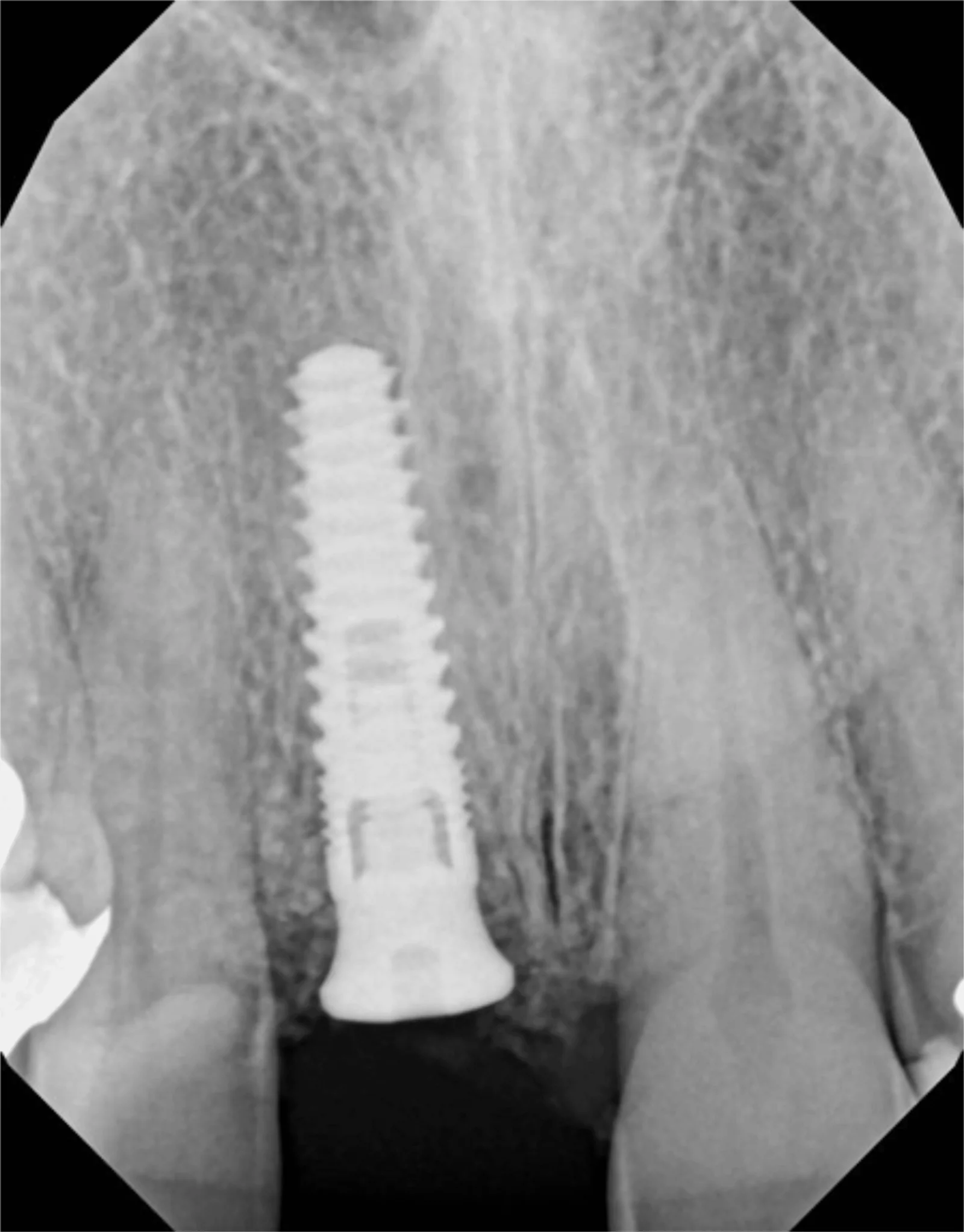
Conventional two-dimensional periapical radiograph of a maxillary central incisor implant following placement. Although the image confirms implant position relative to the adjacent teeth and crestal bone, it cannot accurately assess the buccolingual angulation of the implant within the premaxilla or the thickness of the facial cortical plate. These inherent limitations of two-dimensional imaging may mask facial bone deficiencies, dehiscence, or fenestration that are only reliably detected with three-dimensional imaging.

Implant placement in the esthetic zone requires precise three-dimensional positioning to achieve predictable esthetic and functional outcomes while preserving the facial cortical plate. Although CBCT provides the foundation for treatment planning, the anatomy encountered following tooth extraction may differ from the preoperative scan because of variations in socket morphology, residual septal bone, and facial bone thickness. Conventional periapical radiographs remain an important component of implant placement by confirming mesiodistal positioning relative to adjacent teeth and the crestal bone (Figure 5). However, because they are two-dimensional, they cannot determine the buccolingual position or angulation of the implant, the thickness of the facial plate, or whether the implant is centered within the available alveolar housing.

**Figure 6:**
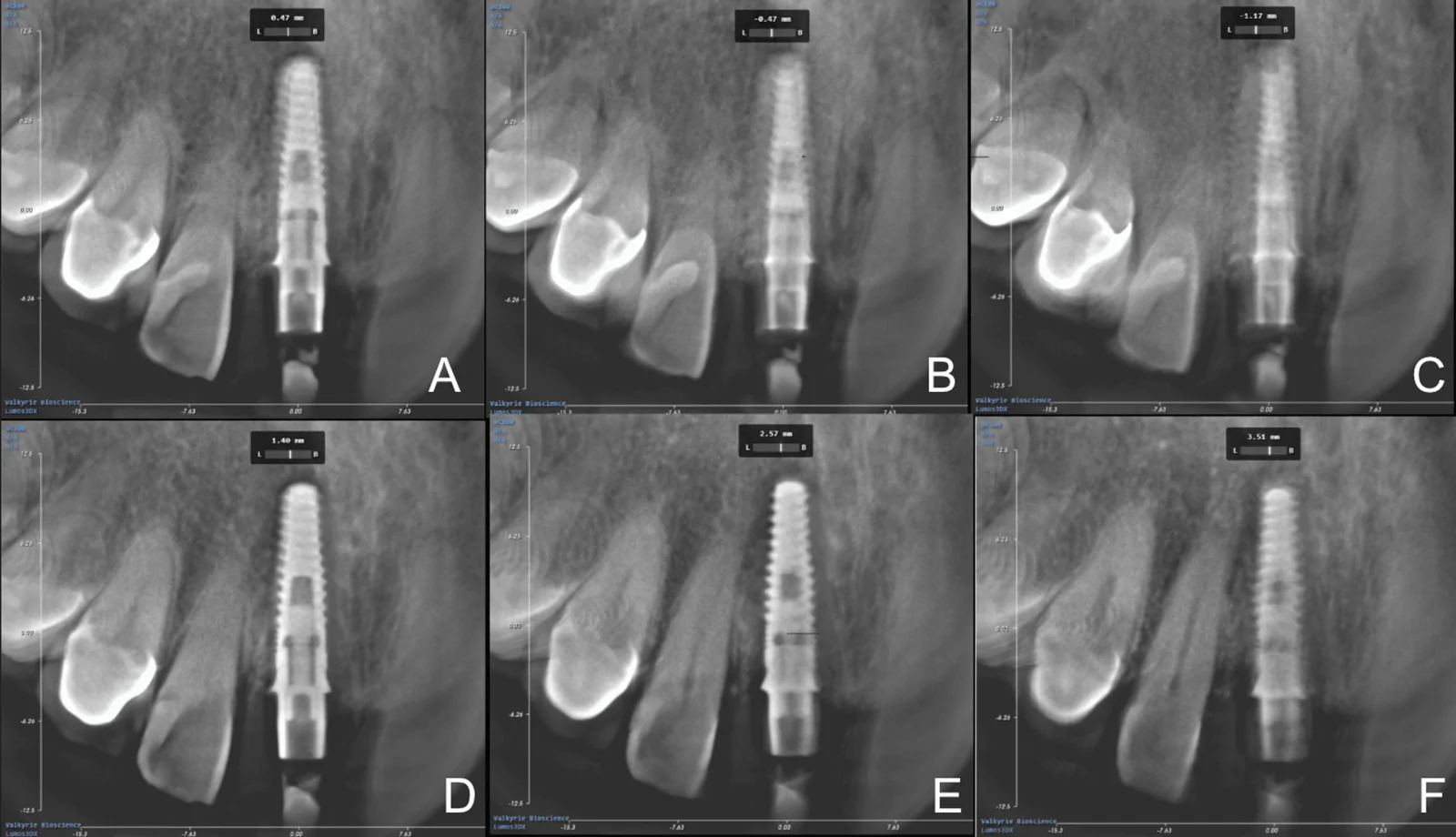
Sequential cross-sectional tomosynthesis images through the anterior maxillary implant site. The numerical values displayed on the images identify the relative position of the reconstructed sections within the tomosynthetic dataset. The sequential sections demonstrate the buccolingual position of the implant within the alveolar housing and its relationship to the facial and palatal cortical plates.

Chairside tomosynthesis with the Lumos 3DX supplements conventional radiography by providing immediate cross-sectional images of the implant site during surgery (Figure 6). These images allow the clinician to visualize the buccolingual position of the implant, evaluate the facial and palatal cortical plates, and confirm that the implant remains centered within the available "triangle of bone." This additional information is not available on a conventional periapical radiograph and provides immediate confirmation that the implant has been positioned as planned. If excessive facial positioning or cortical perforation is identified, corrective action can be taken before completing the procedure, reducing the risk of facial dehiscence, gingival recession, and compromised esthetic outcomes.

Immediate implant placement

Immediate implant therapy presents unique anatomical challenges because extraction socket morphology frequently differs from the appearance seen on preoperative imaging. Remaining septal bone, facial plate thickness, and apical engagement become evident only after the tooth has been removed.

Immediate implant placement may therefore represent a promising application of chairside tomosynthesis. Once the tooth has been removed, the clinical anatomy occasionally differs from the preoperative CBCT, and supplemental sectional imaging may provide additional confidence before the implant is fully seated.

Focused tomosynthesis images may provide additional confirmation that implant positioning remains consistent with restorative objectives while preserving adequate surrounding bone. This information may be particularly valuable in molar extraction sites where socket anatomy can be difficult to appreciate clinically.

Posterior maxillary implant surgery and sinus augmentation

The posterior maxilla often presents limited vertical bone height, making sinus augmentation a common component of implant therapy. CBCT remains essential for evaluating sinus anatomy before treatment; however, intraoperative questions frequently differ from those encountered during treatment planning.

Posterior maxillary implant surgery and sinus augmentation may also represent procedures in which chairside tomosynthesis offers potential clinical value. Immediate confirmation of implant position relative to the sinus floor or grafted site could allow treatment to proceed with greater confidence while maintaining surgical workflow.

Similarly, during sinus augmentation, the timing of tomosynthesis should be based on the intraoperative question. Focused imaging may be obtained after membrane elevation when its relationship to the sinus anatomy requires further assessment, following graft placement to evaluate the grafted region, or after implant placement to assess implant position relative to the sinus floor and grafted site. Routine imaging at each stage is not suggested; rather, tomosynthesis may be used selectively when additional sectional information has the potential to influence the next clinical decision.

Evaluation of implant integration

**Figure 7:**
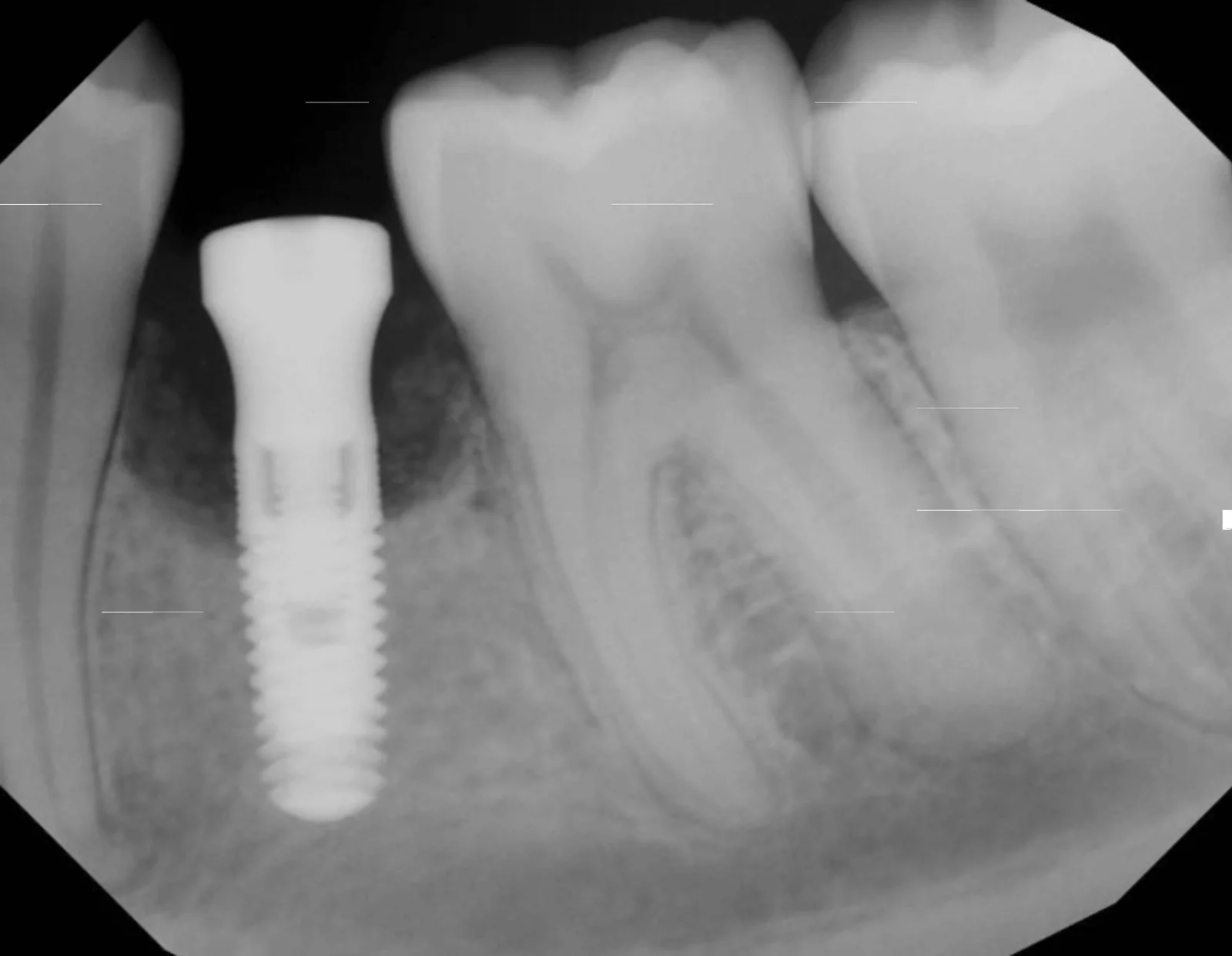
Conventional periapical radiograph of the implant site. The two-dimensional image provides an overall view of the implant and adjacent bone but may obscure facial or lingual peri-implant changes.

Chairside tomosynthesis may also be useful when implant stability or successful osseointegration is clinically uncertain. Implant mobility, percussion, insertion torque, and resonance frequency analysis provide information regarding stability, but they do not directly visualize the bone surrounding the implant. Conventional periapical radiographs may identify crestal bone loss or an obvious peri-implant radiolucency, but their two-dimensional nature can obscure changes on the facial or lingual aspects of the implant (Figure 7).

**Figure 8:**
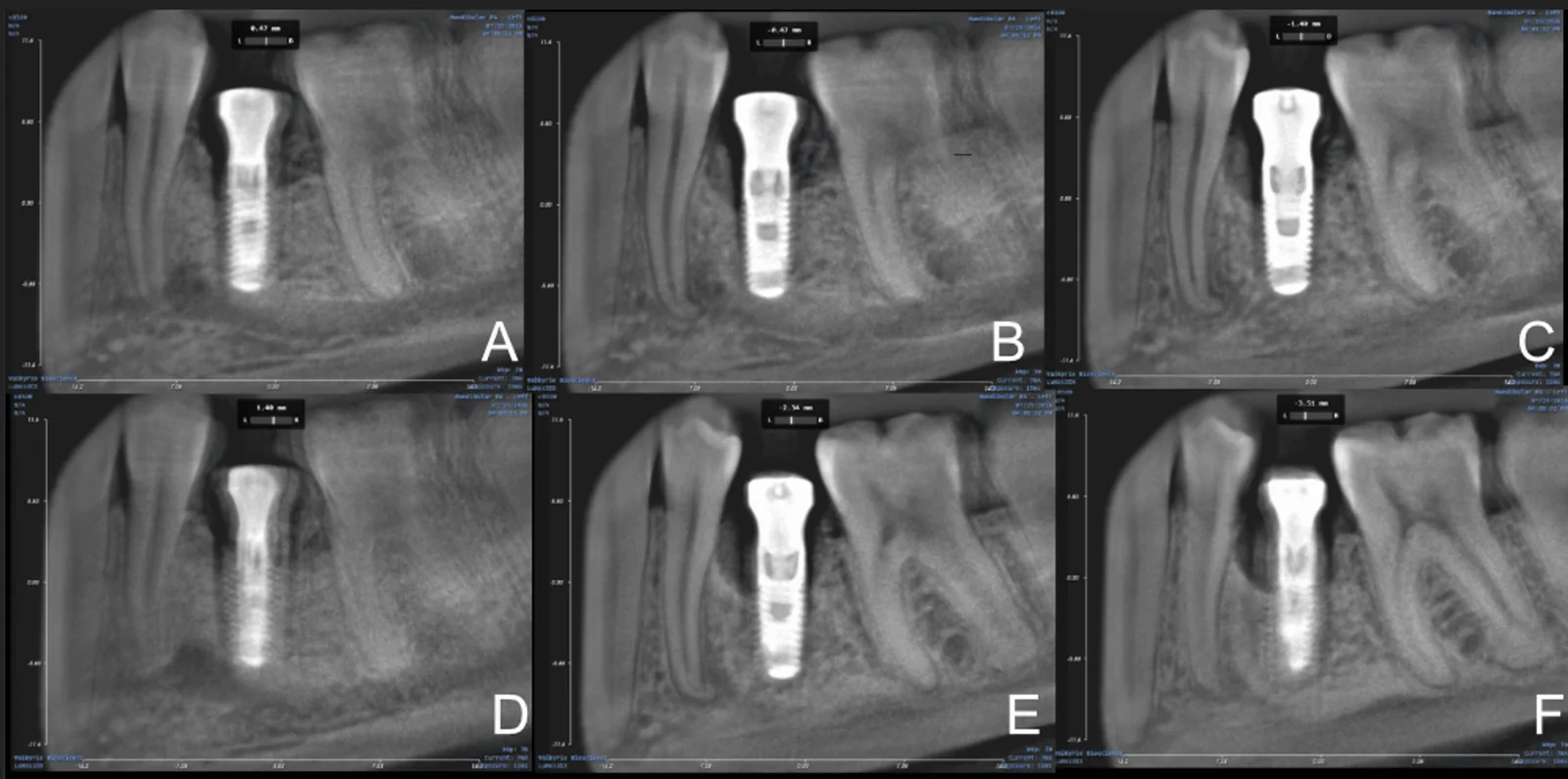
Lumos 3DX tomosynthetic images of the same implant site demonstrating multiple sectional views (A = -0.41, B = -0.49, C = -1.03, D = -1.45, E = -2.54, and F = -3.51) through the implant and surrounding bone, providing information not available on the conventional 2D radiograph (Figure 7).

In comparison, Lumos 3DX provides multiple sectional views through the implant site, allowing the clinician to evaluate the surrounding bone in greater detail (Figure 8). This may help identify localized peri-implant radiolucency, bone loss, or other changes suggesting compromised integration that are not apparent on the conventional 2D image. This additional information can be particularly useful before restorative loading when clinical findings raise questions regarding integration or when an implant demonstrates unexpected mobility or discomfort.

Conversely, visualization of bone surrounding the implant without an evident peri-implant radiolucency can provide additional information supporting the clinical assessment of successful integration. Tomosynthesis does not replace clinical evaluation or other methods of assessing implant stability, but complements those findings by providing improved visualization of the peri-implant bone.

Multiple implants and full-arch rehabilitation

Minor deviations in implant angulation may accumulate during full-arch rehabilitation, particularly when multiple implants are placed sequentially. Confirming implant position during surgery allows small adjustments to be made before they influence subsequent implant placement or prosthetic design.

In these situations, chairside tomosynthesis functions as a decision-support tool rather than a diagnostic examination, providing confidence that treatment remains consistent with the original restorative plan.

Revision surgery and retrieval procedures

Revision procedures often involve altered anatomy, retained implant components, fractured prosthetic screws, or previous grafting materials. Conventional imaging may be limited by superimposition, while metallic artifacts can reduce the diagnostic value of CBCT in localized regions [12-15].

Focused sectional imaging may assist in identifying retained components, confirming retrieval, or documenting implant-related complications without requiring interruption of the procedure.

Clinical situations in which this approach may be useful include posterior mandibular implants close to the inferior alveolar canal, posterior maxillary implants adjacent to the sinus floor, simultaneous sinus augmentation, immediate molar implants, narrow ridges, multiple adjacent implants, full-arch cases, revision procedures, and retrieval of fractured implant prosthetic components. Representative clinical indications for chairside tomosynthesis and the focused intraoperative questions that may be addressed are summarized in Table 2. Potential clinical applications for chairside tomosynthesis include verification of osteotomy trajectory and implant position, assessment of implant relationships to the inferior alveolar canal, maxillary sinus, and cortical plates, evaluation during immediate implant placement and sinus augmentation, confirmation of implant positioning in multiple-implant and full-arch procedures, and localization or confirmation of retrieval of retained or fractured implant components. 

**Table 2:** Suggested clinical applications for chairside tomosynthesis.

Clinical Situation	Intraoperative Question	Potential Benefit	Evidence Level
Posterior mandibular implant	Is implant trajectory appropriate relative to the inferior alveolar canal?	Confirmation before final implant placement	Clinical example in this report
Immediate implant placement	Is implant position consistent with socket anatomy and restorative goals?	Supports implant positioning before final seating	Proposed clinical application
Sinus augmentation	Is implant or graft position satisfactory relative to the sinus floor?	Supplemental confirmation during surgery	Proposed clinical application
Full-arch rehabilitation	Are sequential implant positions maintaining the planned restorative envelope?	Improves confidence before continuing treatment	Proposed clinical application
Revision surgery	Has a retained component been completely retrieved?	Focused localization and documentation	Proposed clinical application
Sedated patient	Can additional imaging be obtained without moving the patient?	Preserves workflow and patient positioning	Proposed clinical application

Workflow advantages

Perhaps the greatest contribution of chairside tomosynthesis is not improved image quality, but improved workflow. Traditional imaging requires the patient to travel to the scanner. Chairside tomosynthesis reverses this relationship by bringing the imaging system directly to the patient.

In the authors' practice, maintaining procedural momentum is an important aspect of efficient implant surgery. Transporting patients for additional imaging may interrupt not only the procedure itself but also the concentration of the surgical team. Chairside imaging has the potential to keep treatment moving while still providing the additional information needed for confident decision-making.

This seemingly simple change has meaningful clinical implications. Surgical instruments remain organized, the operative field is maintained, assistants continue their normal workflow, and treatment proceeds without the disruption associated with patient transport. Instead of pausing the procedure to obtain additional imaging, clinicians can acquire focused sectional information within the operatory and immediately continue treatment. Avoiding transport to a dedicated imaging suite and subsequent patient repositioning may reduce the time required for supplemental intraoperative imaging. However, the magnitude of this potential time savings was not measured in the present report and will vary according to office configuration, imaging workflow, and patient factors.

This approach changes the role of imaging during implant surgery. Rather than serving exclusively as a planning tool, imaging becomes an active component of real-time surgical decision-making.

Importantly, chairside tomosynthesis is not expected to be used routinely during every implant procedure. Its greatest value occurs when a focused clinical question arises that has the potential to alter the next surgical step. Used selectively in this manner, it complements existing imaging protocols while preserving efficiency and procedural continuity.

Imaging during sedation

The workflow advantages of chairside imaging become particularly evident when procedures are performed under intravenous or oral sedation. Transporting sedated patients outside the operatory requires additional personnel, monitoring equipment, and careful attention to patient safety. Even in well-designed surgical practices, moving a sedated patient to obtain additional CBCT imaging interrupts treatment and increases procedural complexity.

Chairside tomosynthesis minimizes these disruptions by allowing supplemental imaging to be acquired while the patient remains comfortably positioned in the dental chair. This reduces unnecessary movement, maintains continuity of patient monitoring, and allows the surgical team to remain focused on treatment. Practical considerations remain important when chairside tomosynthesis is used during sedation. Patient positioning must permit proper alignment of the imaging system while maintaining appropriate physiologic monitoring and access to the patient. Monitoring equipment, intravenous lines, surgical instrumentation, and other devices should be positioned so that they do not interfere with image acquisition or patient management. The surgical team must also maintain appropriate radiation-safety protocols and preserve the operative field during imaging. These considerations should be incorporated into the surgical workflow before chairside imaging is performed in a sedated patient.

Although these advantages may improve efficiency in all implant procedures, they may be particularly meaningful during lengthy surgical appointments involving multiple implants, full-arch rehabilitation, or sinus augmentation. This advantage may become particularly important during procedures performed under intravenous sedation, where avoiding unnecessary patient movement may improve efficiency for both the surgical team and the patient.

Documentation and communication

Images obtained during implant surgery provide benefits that extend beyond the immediate procedure [16]. Intraoperative documentation establishes a visual record of implant position, anatomical relationships, and important clinical decisions made during treatment. These images may assist communication between the surgical and restorative teams, facilitate laboratory discussions, and provide objective documentation for insurance carriers when clinically indicated.

Chairside tomosynthesis may also improve communication with patients. Unexpected findings encountered during surgery can often be explained more effectively when supported by sectional imaging obtained during the procedure. This may enhance patient understanding and reinforce confidence in clinical decision-making.

Chairside tomosynthesis may also be used to establish baseline postoperative images and document key stages of treatment. These images may facilitate communication with restorative colleagues while providing valuable documentation for long-term maintenance and patient education. As implant therapy becomes increasingly interdisciplinary, documentation acquired at critical treatment stages contributes not only to record-keeping but also to continuity of care.

## Discussion

CBCT fundamentally changed implant dentistry by providing clinicians with comprehensive three-dimensional visualization before surgery begins. Chairside tomosynthesis addresses a different phase of treatment by extending sectional imaging into the operative environment.

These technologies should not be viewed as competitors. Each was developed to answer different clinical questions. CBCT remains the preferred modality for comprehensive diagnosis, treatment planning, and evaluation of complex anatomical relationships. Chairside tomosynthesis provides focused supplemental information when additional visualization is needed during surgery. The complementary roles of conventional radiography, CBCT, and chairside tomosynthesis throughout the various phases of implant therapy are summarized in Table 3.

**Table 3:** Proposed imaging continuum in implant dentistry. CBCT: cone-beam computed tomography.

Phase of Care	Primary Clinical Objective	Preferred Imaging
Initial evaluation	Detect pathology and assess patient needs	Conventional radiographs and CBCT
Treatment planning	Comprehensive three-dimensional evaluation	CBCT
Implant surgery	Answer focused intraoperative questions	Chairside tomosynthesis (when indicated)
Postoperative assessment	Verify implant position and establish baseline	Conventional radiographs ± tomosynthesis when clinically indicated
Long-term maintenance	Monitor peri-implant health	Conventional radiographs

This distinction is important because the imaging requirements before surgery are fundamentally different from those encountered during treatment. Comprehensive diagnosis requires a complete anatomical assessment, whereas intraoperative imaging typically aims to confirm a single relationship before proceeding.

Although directly comparable studies evaluating chairside tomosynthesis for intraoperative implant imaging are not currently available, previous investigations of dental tomosynthesis provide relevant background for the clinical applications described in this report. Early work with tuned-aperture computed tomography (TACT), a related limited-angle tomographic technique, demonstrated that sectional dental imaging can reduce the anatomical superimposition inherent in conventional two-dimensional radiography and provide additional information regarding dentoalveolar structures [7,8]. Subsequent clinical evaluation of digital dental tomosynthesis similarly demonstrated visualization of anatomical structures in multiple planes with reduced superimposition compared with conventional intraoral radiography. These findings provide a basis for the present clinical use of chairside tomosynthesis when a focused three-dimensional relationship cannot be adequately assessed on a conventional periapical image.

Previous studies have also investigated tomosynthetic imaging for assessment of osseous changes. Nair et al. reported that TACT provided improved evaluation of osseous healing compared with conventional radiographic imaging, with reconstructed TACT images demonstrating a strong relationship with histomorphometric findings [9,10]. Additional experimental work comparing TACT with conventional computed tomography demonstrated a strong correlation between the two modalities in evaluating the healing of osseous defects. Although these studies did not involve dental implants or the Lumos 3DX system, they support the broader concept that sectional tomosynthetic imaging can provide information regarding osseous structures that may be less apparent on conventional two-dimensional radiographs.

These findings should be interpreted cautiously when applied to the present clinical applications. The current report is intended to describe workflow and potential clinical uses rather than establish diagnostic superiority or improved treatment outcomes. Nevertheless, the existing tomosynthesis literature provides a reasonable technical and diagnostic foundation for using focused sectional imaging to supplement conventional radiography when an intraoperative question arises. Prospective clinical studies comparing chairside tomosynthesis with conventional radiography and CBCT will be necessary to determine diagnostic accuracy, radiation-dose considerations, effect on clinical decision-making, and whether its use ultimately influences implant treatment outcomes. Cost and accessibility should also be considered, as chairside tomosynthesis requires dedicated imaging equipment that may not be available or economically practical in all dental practices, potentially limiting widespread adoption.

Several practical considerations should be taken into account when incorporating chairside tomosynthesis into implant surgery. Radiation exposure should be considered relative to the diagnostic information required, and supplemental imaging should be justified by a specific clinical question rather than obtained routinely. The cost-effectiveness of chairside tomosynthesis has not yet been established and will depend on equipment costs, frequency of use, practice workflow, and whether the additional imaging influences clinical management. Appropriate training is also required for patient positioning, image acquisition, reconstruction, and interpretation, with a learning curve anticipated as clinicians become familiar with tomosynthetic datasets. Finally, the convenience of chairside imaging should not lead to unnecessary imaging; its use should remain selective and based on whether the additional information has the potential to influence the next clinical decision.

The greatest contribution of chairside tomosynthesis may therefore be conceptual rather than technical. It changes imaging from a preoperative event into an intraoperative resource that supports clinical decision-making in real time.

Although clinical experience with chairside tomosynthesis is still developing, the authors view the technology as a natural extension of modern implant imaging rather than a replacement for CBCT. They expect its greatest value will be in answering focused intraoperative questions while allowing clinicians to maintain their normal surgical workflow.

As additional clinical experience is gained, future applications will likely extend beyond implant surgery into endodontics, oral surgery, periodontics, and other areas where focused sectional imaging may improve patient care.

## Conclusions

CBCT has transformed implant dentistry by making comprehensive three-dimensional diagnosis and treatment planning routinely available. Chairside tomosynthesis may complement CBCT by extending focused sectional imaging into the surgical phase of care when additional information is needed to address a specific intraoperative question. By allowing supplemental three-dimensional imaging to be obtained directly within the operatory, this approach has the potential to reduce disruption of the surgical workflow while providing additional information that may assist intraoperative decision-making.

The clinical applications presented in this report illustrate the potential role of chairside tomosynthesis but should be interpreted within the limitations of the currently available evidence. Directly comparable prospective clinical studies are needed to evaluate diagnostic accuracy, radiation exposure, effects on clinical decision-making and workflow, and implant treatment outcomes. Chairside tomosynthesis should therefore be considered a focused adjunct to established imaging protocols rather than a replacement for comprehensive CBCT assessment.
